# Mortality, Falls, and Fracture Risk Are Positively Associated With Frailty: A SIDIAP Cohort Study of 890 000 Patients

**DOI:** 10.1093/gerona/glab102

**Published:** 2021-04-22

**Authors:** Robert Middleton, Jose Luis Poveda, Francesc Orfila Pernas, Daniel Martinez Laguna, Adolfo Diez Perez, Xavier Nogués, Cristina Carbonell Abella, Carlen Reyes, Daniel Prieto-Alhambra

**Affiliations:** 1 Nuffield Department of Orthopaedics, Rheumatology and Musculoskeletal Sciences, Botnar Research Centre, University of Oxford, UK; 2 CIBERFes, Instituto Carlos III, Madrid, Spain; 3 Gerència Territorial de Barcelona, Institut Català de la Salut, Spain; 4 Fundació Institut Universitari per a la recerca a l’Atenció Primària de Salut Jordi Gol i Gurina (IDIAPJGol), Barcelona, Spain; 5 Musculoskeletal Research Unit, IMIM-Hospital del Mar, Barcelona, Spain; 6 Internal Medicine Department, IMIM-Hospital del Mar, Barcelona, Spain; 7 Universitat Autònoma de Barcelona, Bellaterra (Cerdanyola del Vallès), Spain

**Keywords:** Epidemiology, Frailty, Risk factors

## Abstract

**Background:**

Frail subjects are at increased risk of adverse outcomes. We aimed to assess their risk of falls, all-cause mortality, and fractures.

**Method:**

We used a retrospective cohort study using the Sistema d’Informació per al Desenvolupament de l’Investigació en Atenció Primària database (>6 million residents). Subjects aged 75 years and older with ≥1 year of valid data (2007–2015) were included. Follow-up was carried out from (the latest of) the date of cohort entry up to migration, end of the study period or outcome (whichever came first). The eFRAGICAP classified subjects as fit, mild, moderate, or severely frail. Outcomes (10th revision of the International Classification of Diseases) were incident falls, fractures (overall/hip/vertebral), and all-cause mortality during the study period. Statistics: hazard ratios (HRs), 95% CI adjusted (per age, sex, and socioeconomic status), and unadjusted cause-specific Cox models, accounting for competing risk of death (fit group as the reference).

**Results:**

A total of 893 211 subjects were analyzed; 54.4% were classified as fit, 34.0% as mild, 9.9% as moderate, and 1.6% as severely frail. Compared with the fit, frail had an increased risk of falls (adjusted HR [95% CI] of 1.55 [1.52–1.58], 2.74 [2.66–2.84], and 5.94 [5.52–6.40]), all-cause mortality (adjusted HR [95% CI] of 1.36 [1.35–1.37], 2.19 [2.16–2.23], and 4.29 [4.13–4.45]), and fractures (adjusted HR [95% CI] of 1.21 [1.20–1.23], 1.51 [1.47–1.55], and 2.36 [2.20–2.53]) for mild, moderate, and severe frailty, respectively. Severely frail had a high risk of vertebral (HR of 2.49 [1.99–3.11]) and hip fracture (HR [95% CI] of 1.85 [1.50–2.28]). Accounting for competing risk of death did not change results.

**Conclusion:**

Frail subjects are at increased risk of death, fractures, and falls. The eFRAGICAP tool can easily assess frailty in electronic primary care databases in Spain.

The global population is increasing in size and age; United Nations estimates that 1 in 6 individuals will be older than 65 years by 2050, rising to 1 in 4 for North America and Europe (1). Aging populations are expected to place increased pressure on health care systems, which will need to support a greater number of patients with chronic conditions. Frailty, as a measure of vulnerability to ill health, has received increasing attention in recent years; the early identification of frail subjects would help design specific preventive strategies for this population at risk.

Frailty is a concept that recognizes deterioration of multiple body systems and failure of homeostatic mechanisms. Frail individuals are at greater risk of adverse outcomes (higher mortality, loss of independence, complications, and prolonged recovery), and these may result from relatively minor insults (2–4). Identification of frailty can be achieved with phenotypic models or cumulative deficit models (2,5). Phenotypic frailty is determined by clinical assessment for the presence of signs such as slow gait, weak grip, or weight loss. Cumulative deficit models determine frailty by the presence of defined conditions, as identified in the models’ development. These cumulative deficit models generate a frailty index, with higher values indicating greater degrees of frailty. Compared to the phenotypic assessment of frailty, frailty indexes can be implemented in electronic health care records (EHRs), are less time-consuming, and could be used to screen for frailty at a population level (6). The electronic Frailty Index (eFI) was developed using primary care data in the United Kingdom and incorporates 36 potential deficits. Increasing levels of frailty (categorized as mild, moderate, and severe) demonstrated increased mortality, hospital admission, and nursing home admission risks (7). The eFI has subsequently been modified to create the eFRAGICAP for compatibility with Catalonian primary care data, with validation work confirming good discriminatory capacity (8).

Frailty prevalence is dependent on the population being studied and the frailty assessment method used. Collard et al. reported a wide range (4.0%–59.1%) in their 2012 systematic review, which included multiple frailty measures across countries (with cohorts ranging in size from 230 to 8 914) (9). In the development of the eFI, an external validation using 516 007 subjects from the United Kingdom demonstrated a frailty prevalence of 37%, 16%, and 4% for mild, moderate, and severe frailty, respectively (7). Despite variations in frailty prevalence, a meta-analysis of 19 studies (ranging in size from 754 to 36 306, across multiple countries) reported increased mortality risk with increasing frailty index (10).

Fragility fractures are fractures occurring following low-energy trauma and are associated with increased morbidity and mortality (11–13). Management of such injuries is a growing challenge for health care systems, with significant economic consequences. For 2005, the cost of managing osteoporosis-related fractures in the United States was estimated at $17 billion, and for the United Kingdom in 2000 this figure was $1.8 billion, and these figures are expected to rise (14,15). In a study of 6 724 females aged 65 years and older, increasing phenotypic frailty was associated with increased risk of hip fracture, non-spine fracture, and mortality with a 9-year follow-up (16). There is a scarcity of reports using large national data sets investigating associations between fragility fractures and frailty. Given the increasing use of frailty measures to identify vulnerable patients, and the growing population at risk of fragility fractures, a contemporary investigation of these factors is of value.

The aim of this study was to describe the prevalence of frailty among old subjects in Catalonia using the eFRAGICAP, and to determine associations between mortality, fracture risk, and frailty using a large national health care database.

## Materials and Methods

### Study Design, Data Source, and Population

We conducted a retrospective cohort study using data from the Sistema d’Informació per al Desenvolupament de l’Investigació en Atenció Primària (SIDIAP) database (http://www.sidiap.org). The SIDIAP database comprises anonymized electronic medical records of over 5 million patients attending primary care centers in Catalonia (>80% of the population). Information collected included sociodemographic information (year of birth, gender, country of origin, socioeconomic status), clinical measurements (height, weight, waist circumference, blood pressure), recorded diagnoses gathered through 10th revision of the International Classification of Diseases (ICD-10) codes as well as pharmacy invoicing data and hospital admissions (17).

### Study Period, Participants, Inclusion/Exclusion Criteria, and Follow-up

The study period was from January 1, 2007 to the December 31, 2015. Eligible patients were all subjects of at least 75 years old during the study period with at least 1 year of valid data previous to the eFRAGICAP calculation (index date). There were no exclusion criteria. Follow-up was carried out from the latest of date of cohort entry (whenever the subjects were eligible) or the start of the study period, until transfer out of the catchment area, end of the study period or study outcome (death, fractures) whichever came first.

### Exposure

Subjects’ frailty was the main exposure which was calculated for all eligible subjects at the index date with the eFRAGICAP tool (8). The eFRAGICAP is a validated and adapted tool based on the eFI from the United Kingdom (7), which comprises a total of 36 deficits based on the Rockwood model of frailty including medical and pharmacy information (ICD-10, ATC codes, etc.) registered in primary care records. Categories of fit, mild frailty, moderate frailty, and severe frailty were defined by quartiles using the 99th centile as the upper limit as in the eFI study (7). All individual deficit, deficit count, and frailty categories were determined for the study population (Supplementary Table 1).

### Study Outcomes

The outcomes of interest were incident falls, all-cause mortality, and incident fractures: (i) all fractures (excluding face, skull, and digits), (ii) vertebral fractures, and (iii) hip fractures. All outcomes were ascertained during the study period and identified using ICD-10 codes (Supplementary Table 2).

### Confounders

Confounders associated with both the risk of fractures and frailty were predefined a priori. These were the age of the subject at the index date, sex, and socioeconomic status measured with the MEDEA index tool (18).

### Ethical Considerations

This study was approved on the February 28, 2018 by the Scientific and Ethical Committees of SIDIAP (DE-020-Certificat CEIC project nº P17/209).

### Statistical Analyses

Baseline characteristics of participants were described using mean and standard deviation, or median and interquartile range if the distribution of the data was asymmetric, for continuous variables and frequencies for categorical variables. Potential confounders considered for adjustment were those known relevant for frailty and fractures such as age, sex, and socioeconomic status. These confounders were measured at baseline. The Cox proportional hazards regression models were fitted to estimate hazard ratios (HRs) and 95% confidence intervals (95% CIs) adjusted for age, sex, and socioeconomic status taking the fit group as the reference group. Adjusted and unadjusted cause-specific HRs were calculated using cause-specific Cox models for the risk of fractures (all, hip, and vertebra) and the risk of falls accounting for each event for the competing risk of death. Statistical significance was defined at the *p* <.05 level. All statistical analyses were conducted using R version 3.4.5 for Windows using the mstate package and SPSS version 22.

## Results

Baseline characteristics of the population analyzed are reported in Table 1. A total of 893 211 subjects were included and analyzed. Most were old women (mean age of 79 years), rather healthy (with 0 or 1 deficits) coming from an urban setting. The population studied was evenly distributed across the socioeconomic strata. When calculating the frailty index with the eFRAGICAP tool, the majority of subjects included were classified as fit or having mild frailty (54% and 34%, respectively).

**Table 1.** Baseline Characteristics of the Population Analyzed

Characteristics			
Sex	Total	Female	Male
*N* (%)	893 211 (100)	533 457 (59.73)	359 754 (40.27)
Age (y)			
Mean (*SD*)	78.90 (5.23)	79.38 (5.54)	78.17 (4.65)
Median (IQR)	76.08 (75−81.75)	76.75 (75–82.67)	75.17 (75–80.33)
No. of accumulated deficits			
Median (IQR)	1 (0–3)	1 (0–3)	1 (0–3)
Frailty category^a^, *n* (%)			
Fit	486 683 (54.49)	291 415 (54.63)	195 268 (54.28)
Mild	303 802 (34.01)	179 578 (33.66)	124 224 (34.53)
Moderate	88 473 (9.91)	53 491 (10.03)	34 982 (9.72)
Severe	14 253 (1.60)	8 973 (1.68)	5 280 (1.47)
Habitat, *n* (%)			
Rural	181 731 (20.35)	104 933 (19.67)	76 798 (21.35)
Urban	709 660 (79.45)	427 337 (80.11)	282 323 (78.48)
Missing	1 820 (0.20)	1 187 (0.22)	359 121 (0.18)
MEDEA^b^, *n* (%)			
U1	138 565 (19.53)	86 554 (20.25)	52 011 (18.42)
U2	117 648 (16.58)	71 109 (16.64)	46 539 (16.48)
U3	112 573 (15.86)	66 899 (15.65)	45 674 (16.18)
U4	106 135 (14.96)	62 153 (14.54)	43 982 (15.58)
U5	91 270 (12.86)	53 395 (12.49)	37 875 (13.42)
Missing	143 469 (20.22)	87 227 (20.41)	56 242 (19.92)

*Notes*: IQR = interquartile range.

^a^Fit = 0 or 1 deficit, mild = 2–4 deficits, moderate = 5–8 deficits, and severe = ≥9 deficits. ^b^MEDEA = social deprivation score being U1 the least deprived and U5 the most deprived.

Patients were followed for 7.23 years (95% CI: 7.23–7.24), 8.30 (8.30–8.31), and 8.63 (8.63-8.63) for the outcomes of mortality, fractures, and falls. Incidence rates per 1 000 person-year (95% CI) was 55.82 (55.33–56.07) for mortality, 18.81 (18.53–18.96) for fractures, and 10.98 (10.76–11.09) for falls.

**Figure 1.**
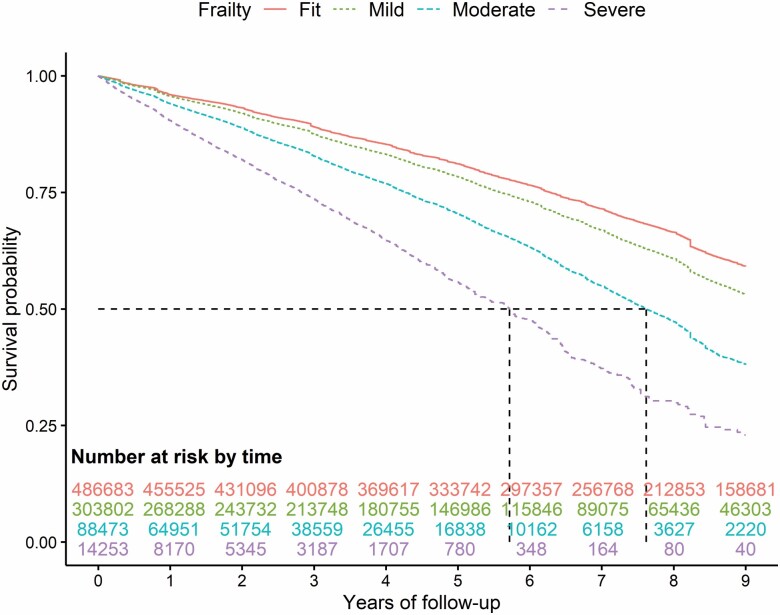
Nine-year Kaplan–Meier survival curve for the outcome of mortality according to the variable frailty.

**Figure 2.**
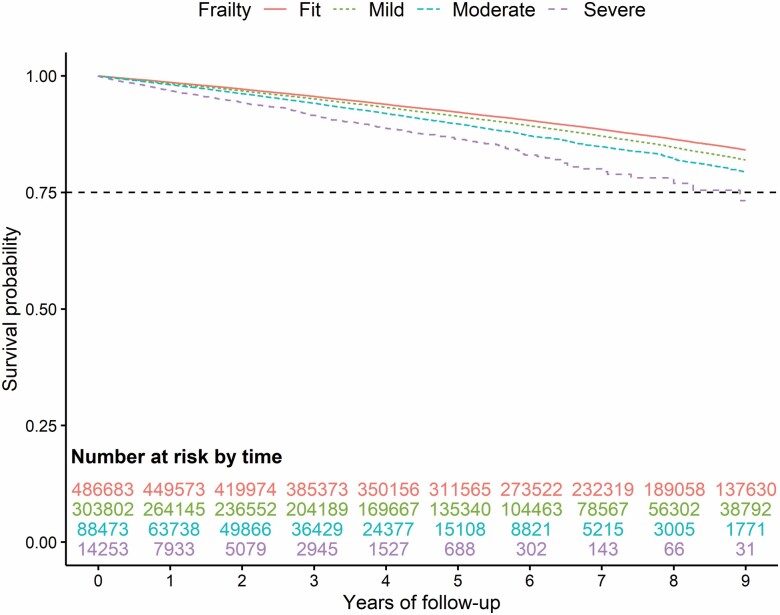
Nine-year Kaplan–Meier survival curve for the outcome of fracture according to the variable frailty.

**Figure 3.**
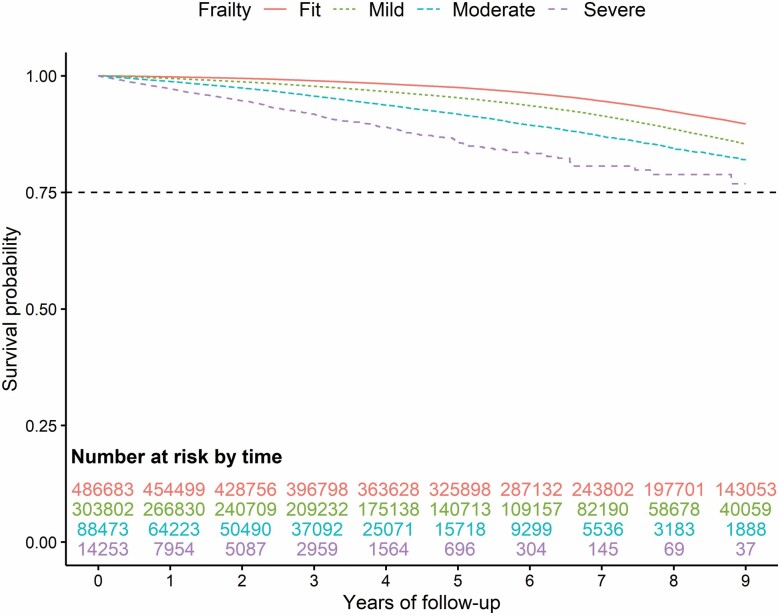
Nine-year Kaplan–Meier survival curve for the outcome of falls according to the variable frailty.

Kaplan–Meier survival curves for the outcomes of mortality, fractures, and falls are reported in Figures 1–3. During the follow-up, mortality showed the greatest increasing trend with increasing frailty (especially pronounced for those classified as moderate or severe frailty) compared to those with mild or those classified as fit (Figure 1). To a lesser degree, the same trend is seen for overall fractures (Figure 2; Supplementary Figure 1) and falls (Figure 3) with a greater incidence of outcomes among those with moderate and severe frailty.

Cox regression models for the unadjusted and adjusted association between frailty and the outcomes of interest (death, fractures, and falls) are reported in Table 2. After adjusting for potential confounders and compared with the fit subjects (reference group), the risk of falling nearly doubled with every increase in frailty (adjusted HR of 1.55, 2.74, and 5.94 for mild, moderate, and severe frailty, respectively). Mortality also showed the same increasing trend, with moderate and severe frailty reporting 2- and 4-fold increased risk of death compared to the fit group. Regarding fractures, overall fracture risk also increased with increasing frailty (adjusted HR of 1.21, 1.51, and 2.36 for mild, moderate, and severe frailty, respectively). This was also true for individual fracture categories; compared to fit subjects, subjects with severe frailty seemed to have a greater risk of vertebral and hip fracture (adjusted HR of 2.49 and 1.85, respectively). Cause-specific Cox analysis accounting for the competing risk of death (Supplementary Tables 3 and 4) were in line with the previous results.

**Table 2.** Association Between Frailty and the Risk of Death, Fractures, and Falls

	Mortality			Fractures (all)			Vertebral Fracture			Hip Fracture			Falls		
	Number of Events	HR	95% CI	Number of Events	HR	95% CI	Number of Events	HR	95% CI	Number of Events	HR	95% CI	Number of Events	HR	95% CI
Nonadjusted															
Fit (ref. *p* < .001)	162 112	1.00	(reference)	486 683	1.00	(reference)	3 812	1.00	(reference)	7 504	1.00	(reference)	28 593	1.00	(reference)
Mild frailty	87 412	1.19	1.181.20	303 802	1.14	1.121.15	2 293	1.29	1.221.37	3 559	1.00	0.971.05	18 508	1.57	1.541.60
Moderate frailty	19 620	1.72	1.691.75	88 473	1.35	1.311.38	535	1.82	1.662.00	680	1.14	1.051.23	4 332	2.80	2.702.89
Severe frailty	2 972	2.77	2.682.88	14 253	1.97	1.842.12	81	2.85	2.283.56	89	1.48	1.201.82	757	6.13	5.706.59
Total deficit count	—	1.10	1.091.10	—	1.06	1.061.07	—	1.12	1.101.13	—	1.02	1.011.03	—	1.22	1.211.22
Adjusted															
Fit (ref. *p* < .001)		1.00	(reference)		1.00	(reference)		1.00	(reference)		1.00	(reference)		1.00	(reference)
Mild frailty	—	1.36	1.351.37	—	1.21	1.201.23	—	1.23	1.171.29	—	1.11	1.061.15	—	1.55	1.521.58
Moderate frailty	—	2.19	2.162.23	—	1.51	1.471.55	—	1.67	1.521.83	—	1.32	1.221.43	—	2.74	2.662.84
Severe frailty	—	4.29	4.134.45	—	2.36	2.202.53	—	2.49	1.993.11	—	1.85	1.502.28	—	5.94	5.526.40
Total deficit count	—	1.16	1.151.16	—	1.09	1.081.09	—	1.10	1.091.11	—	1.05	1.041.07	—	1.21	1.211.22

*Note*: HR = hazard ratio.

Sensitivity analyses were carried out in men and women separately; adjusted Cox regression models found an overall higher risk of suffering all outcomes (death, fractures, and falls) with increasing frailty compared to the fit category in both men and women. Men with frailty (mild, moderate, and severe) had a higher risk of death, fractures, and falls compared to women (Supplementary Table 5). Cause-specific Cox analysis accounting for the competing risk of death (Supplementary Table 6) did not change the results.

## Discussion

In this large longitudinal population-based cohort study, we found that increasing levels of frailty (defined by eFRAGICAP) were associated with increased risks of mortality, fractures, and falls. Subjects with moderate and severe frailty had over 2- and 4-fold increased risk of death compared with those classified as fit. Regarding the risk of fractures, and compared to fit subjects, those with moderate and severe frailty had an increased risk of vertebral fractures (HR of 1.67 and 2.49, respectively) and hip fractures (HR 1.32 and 1.85). Total eFRAGICAP deficit count also demonstrated a positive association with mortality and fracture risk.

Many frailty instruments have been developed in recent years; however, few are the ones that assessed frailty on EHRs (7,19–22). The prevalence of frailty within the Catalonian population, as defined by the eFRAGICAP, was similar to that of the UK population, as defined by the eFI in Clegg et al.’s validation study. Of the study populations, the majority were predominantly fit or mildly frail (80% for eFI and 89% for eFRAGICAP), with moderate (16% vs 10%, respectively) and severe frailty (4% vs 2%, respectively) less prevalent (7). Recently, the eFI was also adapted to an EHR in the United States and calculated for 12 798 patients (22), with 40.1% of their patients’ classified as frail, which is slightly lower than our results. The adaptation of the eFI in each of the studies, with different codes included depending on their availability in the databases, could explain the different prevalence of frailty found. Further differences with previous reports such as the customized frailty indexes used or the lower number of patients included (19,21) render comparison difficult.

Previous studies carried out in Catalonia (23,24), both using Fried’s criteria in subjects older than 70 and 75 years, demonstrated different prevalence’s for frailty, with 31% frail and 49% prefrail in the Serra-Prats et al.’s investigation (23) and 46.9% prefrailty and 9.6% of frailty in the Escobar-Bravo et al.’s investigation (24). While phenotypic and index-based frailty measures cannot be directly compared, these results are not dissimilar.

Regarding mortality, our estimations were similar to that reported by Clegg et al. (7). At 5 years, the adjusted mortality HR was reported as 1.66, 2.54, and 3.84 for mild, moderate, and severe frailty, respectively. Our population, with 9-year follow-up, demonstrated similar HRs of 1.36, 2.19, and 4.29 respectively. Two of the reports that assessed frailty in EHRs (19,22) and a previous meta-analysis (10) found that frailty was associated with an increased risk of death and another one with an overall risk of adverse outcomes (21), which is also in accordance with our results.

Concerning fracture risk, Ensrud et al. (16) investigated the association between phenotypic frailty and fractures in women aged at least 65 years during 9 years of follow-up and found similar results as ours, despite the different frailty definition used (phenotypic vs cumulative health deficits). We found an adjusted HR of 1.32 for hip fracture in the moderately frail cohort, and an adjusted HR of 1.27 with intermediate frailty. Risk was greater in the severely frail group, at 1.85 versus 1.4 in Ensrud’s cohort (16). Methods of frailty determination, and variables adjusted for, likely contributed to the differences here. Kojima’s meta-analyses of frailty and fracture (any site) risks are also in keeping with these figures (HR of 1.57 for frail and 1.30 for prefrail) (25). Other previous studies that analyzed the association between frailty and vertebral fractures found similar results to ours; a 30% increased risk of vertebral fractures with every 0.10 increase in the frailty index score was reported in the Canadian Multicentre Osteoporosis Study (CaMos study) and this risk was higher for vertebral than for hip fractures (26). As the aforementioned study, we also found an increased risk of vertebral and hip fractures in our severely frail population. However, the increased risk found for vertebral fractures needs to be interpreted with caution, given that we only gathered clinical vertebral fractures (not always radiologically confirmed), unlike hip fractures which usually require hospital care. Vertebral fractures are often underdiagnosed (27) and therefore medical doctors may be more prone to search for them among frail subjects rather than in those who are fit. At last, increasing frailty implies a greater number of comorbidities, which could increase the risk of vertebral fractures on their own and require closer monitoring by the medical doctor with a greater number of visits, leading to a greater number of diagnoses of vertebral fractures.

To our knowledge, this is the first study to use an eFI such as the eFRAGICAP in a large real-world database in Spain. The previous longitudinal studies (23,24) carried out in Spain used the Frieds’ criteria which require large time-consuming questionnaires which are difficult to implement in primary care. Moreover, our study population is one of the largest to date used for the investigation of a frailty index and associations with mortality and fracture risk. The SIDIAP database is a primary care database designed specifically for use as a research database with data quality validation measures, and we have used a frailty index that has been previously validated for use in the population of interest which supports the external validity of our results and the use of this measure in real world. This is reinforced given that our results are similar to previously published figures for the outcomes of interest despite the population differences.

We have demonstrated an increased risk of mortality, fracture, and falls in association with increasing degrees of frailty, as determined using the eFRAGICAP tool. These risks remained after adjustment for confounding variables. Frailty assessment is a valuable method to identify at-risk patients; however, such assessments need to be easy to implement to be able to use them in primary care where time per visit is limited. A frailty tool such as eFRAGICAP which can categorize frailty using routinely collected primary care data (in comparison to phenotypic assessments requiring specific clinical reviews) is of great relevance. The same is seen in the United Kingdom, where frailty measures (eFI) are being actively implemented in primary care in the United Kingdom in all patients over the age of 65. This facilitates targeted interventions to maintain patient health, reducing the risk of unplanned hospital admissions and death. The associations between frailty and unplanned hospital admission and mortality supported the implantation of this process. This study also adds to the value of such assessments, as frailty level can also be used to identify patients at increased risk of fracture (with the attendant morbidity and mortality). Fracture prediction tools (such as the Fracture Risk Assessment Tool—FRAX) are already used to identify patients who may benefit from bone protection therapy. Li et al. compared a frailty index and the FRAX in predicting future risk of fractures, and found similar performance, particularly for high-risk patients (28). The identification of frail patients can, therefore, also be seen as an opportunity to start bone protection, to reduce fracture risk.

From our results, we can speculate that any strategy for reducing the impact of fractures should include not only bone-targeted interventions but also general management of the frailty syndrome. Moreover, since fractures can be the consequence but also the cause of increased frailty, we cannot disentangle both problems in the clinical and public health approach to these patients.

Limitations of our study warrant consideration. In the first place, the identification of frail subjects is complex and involves medical, social, and phenotypical aspects that are not always captured in an electronic database such as the SIDIAP database. Despite that our results with the eFRAGICAP are similar to what has been previously reported in the United Kingdom (7), we cannot exclude the possibility that we might have underestimated the true prevalence of subjects in each frailty category. Second, we did not fully adjust for other potential confounders, such as smoking, alcohol consumption, or physical activity. These were indirectly captured through the deficits of the eFRAGICAP and therefore partially accounted for. Moreover, frail subjects require usually a closer monitorization because of their comorbidities and therefore are more prone to visit the primary care doctors, increasing the possibilities of being diagnosed of an outcome; this could partly explain our increased risk of vertebral fractures. Furthermore, we could only assess clinical vertebral fractures (with no information regarding x-rays), leading to a possible underestimation of the true incidence of vertebral fractures. At last, in spite that the collection of data in the SIDIAP database has been previously validated (17), there is still the possibility of misclassifications, which should be considered.

## Conclusion

Frailty measured by the eFRAGICAP tool is associated with an increased risk of mortality, fractures, and falls in subjects over the age of 75 years. The eFRAGICAP tool can be easily implemented for the assessment of frailty in routinely collected primary care databases in Spain.

## Supplementary Material

glab102_suppl_Supplemental_Material
