# Supplementary material for: Mortality, Falls, and Fracture Risk Are Positively Associated With Frailty: A SIDIAP Cohort Study of 890 000 Patients
Source: J Gerontol A Biol Sci Med Sci. 2021 Apr 22;77(1):148–54. doi: 10.1093/gerona/glab102 (PMC8751782; doi:10.1093/gerona/glab102)

eTable 1: List of items included in the eFRAGICAP tool

	Codes or index/classifications considered to define the deficit
1. Mobility	ICD-10: Z99.3; Barthel index
2. Home care	ICD-10: Z74.*; Barthel index
3. Activity limitation	Dependence code (VZ3001); Lawton and Barthel index, ICD-10: Z73.6
4. Sight problems	ICD-10: E10.3, E11.3, E12.3, E13.3, E14.3, Q12.0, H59.0, H35, H35.*, H36, H36.*, H53.*, H53, H54, H54.*, H26, H26.*, H25, H25.*, H28, H28.*, Z97.3"; retinopathy code (RDMD/RDME)
5. Hearing problems	ICD-10: H90, H90.*, H91, H91.*, Z97.4, Z96.2, Z46.1"
6. Need of cures	ICD-10: Z59.3, Z63.6
7. Social vulnerability	ICD-10: Z63.9, Z63.2, Z63.4, Z60.9, Z59.1, Z59.6, Z73.4, Z73.5, Z60.2, Z60.4, Z60, Z60.*, Z59, Z59.0, Z59.1, Z59.2, Z59.4, Z59.5, Z59.6, Z59.7, Z59.8, Z59.9, Z60.2, Z73.91, Z91.81, Z91.2; Tests of social vulnerability: Gijon, TIRS, Barber, OARS
8. Falls	ICD-10: R29.6, R26.8, W0*, W1*; Number of falls
9. Urinary incontinence	ICD-10: R32, N39.3, N39.4; NANDA nursery codes: I00176, I132, I13211, I13212, I13213, I13214, I13215
10. Weight loss/Anorexia	ICD-10: R63.4, R63.3, R63.0, R64, E46; BMI; Mini Nutrition Assessment test
11. Cognitive impairment	ICD-10: F06.7, F00, F00.*, F01, F01.*, F02, F02.*, F03, F03.*, G30, G30.*, G31, G31.*; Validated MMSE for Spain (Lobo test), Pfeiffer test,
12. Dizziness	ICD-10: R42, R42.*, H81, H81.*, H82, H82.*
13. Dyspnoea	ICD-10: R06.0; clinical codes for dyspnoea: ER1030 and VK4031
14. Polypharmacy	5 or more active prescriptions
15. Sleeping problems	ICD-10: F51, F51.*, G47.0, I621, G47, G47.*
16. Anaemia	ICD-10: D50, D50.*, D46, D46.*, D51, D51.*, D52, D52.*, D53, D53.*, D55, D55.*, D56, D56.*, D57, D57.*, D58, D58.*, D59, D59.*, D60, D60.*, D61, D61.*, D62, D62.*, D63, D63.*, D64, D64.*; Haemoglobin levels
17. Hypertension	ICD-10: I10, I11, I11.0, I11.9, I12, I12.0, I12.9, I13, I13.0, I13.1, I13.2, I13.9, I15, I15.0, I15.1, I15.2, I15.8, I15.9; Systolic and Diastolic blood pressure.
18. Ischemic heart disease	ICD-10: I20, I20.*, I21, I21.*, I22, I22.*, I23, I23.*, I24, I24.*, I25, I25.*

19. Congestive heart insufficiency	ICD-10: I11.0, I13.0, I13.2, I50, I50.*, J81.
20. Cerebrovascular disease	ICD-10: G45, G45.*, G46, G46.*, I60, I60.*, I61, I61.*, I62, I62.*, I63, I63.*, I64, I64.*, I65, I65.*, I66, I66.*, I67, I67.*, I68, I68.*, I69, I69.*, S06.6, S06.5
21. Peripheral vascular disease	ICD-10: I70, I70.*, I71, 71.*, I72, I72.*, I73, I73.*, I74, I74.*, I75, I75.*, I76, I76.*, I77, I77.*, I78, I78.*, I79, I79.*, I80, I80.*, I81, I81.*, I82, I82.*, I83, I83.*, E10.5, E11.5, E12.5, E13.5, E14.5.
22. Atrial fibrillation	ICD-10: I48, I48.*
23. Heart valve disease	ICD-10: I05, I05.*, I06, I06.*, I07, I07.*, I08, I08.*, I39, I39.*, I38, I38.*, I37, I37.*, I36, I36.*, I35, I35.*, I34, I34.*, I33, I33.*
24. Hypotension/syncope	ICD-10: I95.*, R55; Systolic and diastolic blood pressure
25. Diabetes	ICD-10: E10, E10.*, E11, E11.*, E12, E12.*, E13, E13.*, E14, E14.*, H36.0, G59.0, G63.2, H28.0, M14.2, N08.3; Glycosylated haemoglobin levels
26. Feet problems	ICD-10: L84
27. Osteoarthritis	ICD-10 codes: M15, M15.*, M16, M16.*, M17, M17.*, M18, M18.*, M19, M19.*, M00, M00.*, M01, M01.*, M02, M02.*, M03, M03.*, M04, M04.*, M05, M05.*, M06, M06.*, M07, M07.*, M08, M08.*, M09, M09.*, M10, M10.*, M11, M11.*, M12, M12.*, M13, M13.*, M14, M14.*, M42, M42.*, M45, M45.*, Z96.6, M46, M46.*, M47, M47.*, M48, M48.*, M77.2
28. Lung disease	ICD-10: J42, J42.*, J43, J43.*, J44, J44.*, J45, J45.*, J46, I26, I26.*, I27.0, I27.2, I27.9, R05, J96, J96.*, J80, J80.*, J81, J81.*, J84, J84.*, J64, J64.*, J66, J66.*, J67, J67.*, J68, J68.*, J69, J69.*, J70, J70.*
29. Ulcer	ICD-10: K25, K25.*, K26, K27, K27.*, K28, K28.*
30. Thyroid disease	ICD-10: D34, D34.*, R94.6, E01, E01.*, E02, E02.*, E03, E03.*, E04, E04.*, E05, E05.*, E06, E06.*, E07, E07.*
31. Chronic kidney disease	ICD-10: N18, N18.*, N19, N19.*, Q61, Q61.*, E10.2, E11.2, E13.2, E14.2
32. Osteoporosis	ICD-10: M80, M80.*, M81, M81.*, M82, M82.*, M83, M83.1; Densitometry values (T-Score hip/femur)
33. Frailty fracture	ICD-10: M80, M80.*, M84, M84.*, M48.4, M48.5, M49.5, S12, S12.*, S22, S22.*, S32, S32.*, S42, S42.*,

	S52, S52.*, S62, S62.*, S72, S72.*, S82, S82.*, S92, S92.*, T08, T08.0, T10.0
34. Parkinson disease	ICD-10: G20, G20.*, G21, G21.*, G22, G22.*, G25, G25.*, R25.1, Y46, Y46.7, F02.3
35. Urinary system disease	ICD-10: N40, N40.*, N41, N41.*, N42, N42.*, I1322, R30, R30.*, R31, R31.*, R33, R33.*, Y84.6, N0*, N1*, N2*, N30, N30.*, N31, N31.*, N32, N32.*, N33, N33.*, N34, N34.*, N35, N35.*, N36, N36.*, N37, N37.*, N38, N38.*, N39, N39.9
36. Skin ulcers	ICD-10: L98.4, I83.0, I89.0, L97, L89, E10.5, E11.5, E12.5, E13.5, E14.5.

eTable 2: Outcome ICD-10 code list (falls, fractures, all-cause mortality)

Codes ICD – 10 falls					
W03.5	W14	W11.8	W09.7	W02.2	W11.1
W03.1	W14.0	W17.9	W09.8	W02.4	W10.2
W02.6	W14.1	W18.5	W09.2	W00.3	W11.7
W02.7	W14.4	W17.2	W08.6	W00.4	W13.5
W00.6	W14.5	W17.4	W07	W01.0	W13.2
W00.7	W14.7	W17.5	W07.0	W01.1	W12.5
W00.8	W14.8	W11.0	W07.1	W01.2	W12.8
W00.9	W14.9	W10.5	W07.2	W01.3	W15
W01	W14.2	W10	W06.8	W01.4	W18.4
W00.5	W15.0	W10.0	W07.4	W01.5	W18.6
W02.8	W15.4	W10.1	W07.5	W01.6	W17.3
W03	W15.5	W11.2	W06	W01.7	W17.7
W02.5	W15.7	W12	W06.0	W01.8	W19.4
W02	W15.8	W10.6	W06.1	W01.9	W19.1
W02.0	W15.1	W10.7	W06.2	W00.2	W16.8
W00	W15.9	W10.8	W06.3	W03.0	
W00.0	W14.3	W11	W05.9	W02.3	
W00.1	W19.3	W10.3	W06.5	W02.9	
W19.7	W19.5	W09.9	W06.6	W04	
W19.8	W18	W10.4	W04.3	W03.7	
W19.9	W18.0	W09.3	W05	W03.3	
W16.3	W18.1	W09.4	W03.8	W03.6	
W16.4	W18.2	W17.6	W03.9	W06.4	
W16	W18.3	W17.1	W04.0	W05.4	
W15.2	W12.9	W17.8	W05.8	W05.6	
W15.3	W12.1	W16.6	W04.4	W04.7	
W16.9	W12.2	W16.7	W04.5	W04.9	
W17	W12.3	W07.9	W04.6	W13.8	
W17.0	W12.4	W08	W04.8	W16.2	
W16.0	W12.6	W08.0	W06.7	W15.6	
W16.1	W12.0	W07.7	W05.1	W14.6	
W19.6	W13	W08.2	W05.2	W16.5	
W18.8	W13.0	W08.3	W05.3	W07.6	
W18.9	W13.1	W06.9	W05.5	W09	
W19	W13.3	W08.7	W05.0	W09.1	
W19.0	W13.4	W08.8	W05.7	W08.1	
W19.2	W12.7	W08.9	W04.1	W08.4	
W18.7	W11.3	W08.5	W04.2	W07.3	
W13.6	W11.4	W09.0	W03.2	W09.6	
W13.7	W11.5	W07.8	W03.4	W11.9	
W13.9	W11.6	W09.5	W02.1	W10.9	
Codes ICD – 10 fractures					

S72.9	S32.5	S52.5	S22.0	S72.2	
T08.0	M48.4	S32.0	S72.0	S72	
T10.0	S52.6	S32.1	S72.1	S42.2	
Codes ICD – 10 Death					
R96.1	I46.1				

eTable 3. Cause-specific Cox Models analysing the risk of overall fractures and falls among frail subjects accounting for the competing risk of death

	Fractures (All)		Mortality CR (csc)[a]		Falls		Mortality CR (csc)[a]	
Non Adjusted	HR	95% CI	HR	95% CI	HR	95% CI	HR	95% CI
Fit (Ref. p<0.001)								
Mild frailty	1.14	1.12-1.15	1.19	1.18-1.20	1.57	1.54-1.60	1.19	1.18-1.20
Moderate frailty	1.35	1.31-1.38	1.73	1.70-1.76	2.80	2.71-2.89	1.72	1.70-1.75
Severe frailty	1.97	1.84-2.12	2.82	2.71-2.93	6.13	5.70-6.59	2.78	2.68-2.89
Total deficit count	1.06	1.06-1.07	1.10	1.10-1.10	1.22	1.21´1.22	1.10	1.10-1.10
Adjusted	HR	95% CI	HR	95% CI	HR	95% CI	HR	95% CI
Fit (Ref. p<0.001)								
Mild frailty	1.21	1.20-1.23	1.36	1.35-1.38	1.55	1.52-1.58	1.36	1.35-1.37
Moderate frailty	1.51	1.47-1.55	2.24	2.20-2.27	2.74	2.65-2.84	2.20	2.17-2.23
Severe frailty	2.36	2.20-2.53	4.39	4.23-4.56	5.94	5.52-6.40	4.29	4.13-4.45
Total deficit count	1.09	1.08-1.09	1.16	1.16-1.16	1.21	1.21-1.22	1.16	1.15-1.16

a-CR: Competitive risk. (csc): cause-specific

eTabla 4. Cause-specific Cox Models analysing the risk of vertebral and hip fractures among frail subjects accounting for the competing risk of death

	Hip Fracture		Mortality CR (csc)[a]		Vertebral Fracture		Mortality CR (csc)[a]	
Non Adjusted	HR	95% CI	HR	95% CI	HR	95% CI	HR	95% CI
Fit (Ref. p<0.001)								
Mild frailty	1.01	0.97-1.05	1.19	1.18-1.20	1.29	1.22-1.36	1.19	1.18-1.20
Moderate frailty	1.14	1.05-1.23	1.72	1.70-1.75	1.82	1.66-2.00	1.72	1.70-1.75
Severe frailty	1.48	1.20-1.82	2.79	2.68-2.89	2.85	2.28-3.56	2.78	2.68-2.88
Total deficit count	1.02	1.01-1.03	1.10	1.10-1.10	1.12	1.10-1.13	1.10	1.10-1.10
Adjusted	HR	95% CI	HR	95% CI	HR	95% CI	HR	95% CI
Fit (Ref. p<0.001)								
Mild frailty	1.11	1.06-1.15	1.36	1.35-1.38	1.23	1.16-1.29	1.36	1.35-1.37
Moderate frailty	1.32	1.22-1.43	2.20	2.17-2.24	1.67	1.52-1.83	2.19	2.16-2.23
Severe frailty	1.85	1.50-2.28	4.32	4.16-4.48	2.49	1.99-3.11	4.30	4.14-4.46
Total deficit count	1.05	1.04-1.07	1.16	1.15-1.16	1.10	1.09-1.11	1.16	1.15-1.16

a-CR: Competitive risk. (csc): cause-specific

eTable 5. Adjusted Cox-regression models in men and women.

	Mortality				Fractures (All)				Vertebral Fracture				Hip Fracture				Falls			
	Women		Men		Women		Men		Women		Men		Women		Men		Women		Men	
	HR	95%CI	HR	95% CI	HR	95% CI	HR	95%CI	HR	95%CI	HR	95%CI	HR	95%CI	HR	95%CI	HR	95%CI	HR	95%CI
Fit (Ref. p<0.001)																				
Mild frailty	1.34	1.33-1.36	1.36	1.34-1.38	1.20	1.18-1.22	1.26	1.22-1.30	1.24	1.17-1.32	1.21	1.10-1.33	1.08	1.03-1.26	1.24	1.14-1.36	1.55	1.52-1.58	1.58	1.52-1.63
Moderate frailty	2.09	2.04-2.13	2.32	2.26-2.37	1.44	1.40-1.49	1.79	1.69-1.90	1.68	1.51-1.86	1.65	1.36-1.99	1.28	1.17-1.40	1.55	1.29-1.86	2.69	2.59-2.80	2.92	2.74-3.12
Severe frailty	3.91	3.72-4.11	4.79	4.54-5.06	2.17	2.00-2.35	3.20	2.77-3.70	2.54	1.98-3.25	2.19	1.31-3.65	1.69	1.33-2.15	2.66	1.71-4.15	5.54	5.09-6.03	7.28	6.30-8.41

eTabla 6. Cause-specific Cox Models analysing the risk of fractures (overall, vertebral and hip) and falls among frail subjects accounting for the competing risk of death in men and women.

	Fractures (All)		Mortality CR (csc)[a]		Vertebral facture		Mortality CR (csc)[a]		Hip fracture		Mortality CR (csc)[a]		Falls		Mortality CR (csc)[a]	
Adjusted	HR	95% CI	HR	95% CI	HR	95% CI	HR	95% CI	HR	95% CI	HR	95% CI	HR	95% CI	HR	95% CI
Women																
Fit (Ref. p<0.001)																
Mild frailty	1.20	1.18-1.22	1.35	1.33-1.37	1.24	1.17-1.32	1.35	1.33-1.36	1.08	1.03-1.13	1.35	1.34-1.37	1.57	1.54-1.60	1.19	1.18-1.20
Moderate frailty	1.44	1.40-1.49	2.13	2.08-2.17	1.67	1.50-1.86	2.09	2.04-2.12	1.28	1.17-1.40	2.10	2.06-2.14	2.80	2.71-2.89	1.72	1.70-1.75
Severe frailty	2.17	2.00-2.35	3.99	3.79-4.21	2.54	1.98-3.25	3.91	3.72-4.11	1.69	1.33-2.15	3.94	3.74-4.13	6.13	5.70-6.59	2.78	2.68-2.89
Men																
Fit (Ref. p<0.001)																
Mild frailty	1.26	1.22-1.30	1.37	1.35-1.38	1.21	1.10-1.34	1.36	1.35-1.38	1.24	1.14-1.36	1.37	1.35-1.38	1.55	1.52-1.58	1.36	1.35-1.37
Moderate frailty	1.79	1.69-1.90	2.35	2.29-2.40	1.64	1.36-1.99	2.32	2.27-2.38	1.55	1.29-1.85	2.32	2.27-2.38	2.74	2.65-2.84	2.20	2.17-2.23
Severe frailty	3.20	2.77-3.70	4.88	4.62-5.15	2.18	1.31-3.65	4.81	4.56-5.09	2.66	1.71-4.15	4.83	4.57-5.10	5.94	5.52-6.40	4.29	4.13-4.45

eFigure 3: Nine-year Kaplan–Meier survival curve for the outcome of fractures of hip (Left) and fracture of vertebra (Right) according to the variable Frailty

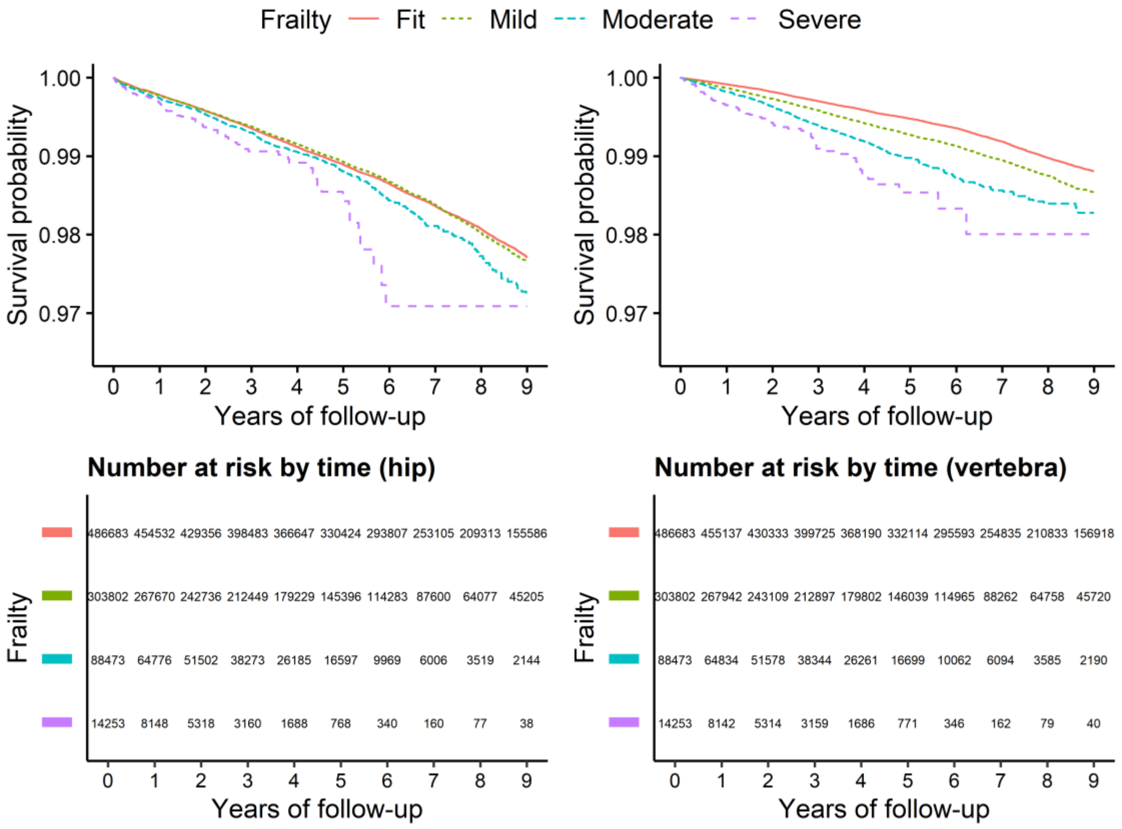

Supplement: glab102_suppl_Supplemental_Material [file glab102_suppl_supplemental_material.pdf]
